# Gratitude and Life Satisfaction among Older Adults in Saudi Arabia: Social Support and Enjoyment of Life as Mediators

**DOI:** 10.3390/bs13070527

**Published:** 2023-06-22

**Authors:** Mogeda El Sayed El Keshky, Shatha Jamil Khusaifan, Feng Kong

**Affiliations:** 1 Department of Psychology, Faculty of Arts and Humanities, King Abdulaziz University, Jeddah 21589, Saudi Arabia; skhusaifan@kau.edu.sa; 2 Department of Psychology, Faculty of Arts, Assiut University, Asyut 71515, Egypt; 3 School of Psychology, Shaanxi Normal University, Xi’an 710062, China; kongfeng87@126.com

**Keywords:** gratitude, enjoyment of life, life satisfaction, social support, aging, older adults, Saudi Arabia

## Abstract

This cross-sectional study aimed to investigate the relationship between gratitude and life satisfaction, and the mediation role of social support and enjoyment of life among older individuals. The measures employed include the Gratitude, Resentment, and Appreciation test; the Enjoyment of Life scale; the Satisfaction with Life scale; and the Multidimensional Scale of Perceived Social Support. These were administered to a sample of 260 older individuals aged between 60 and 80. The main findings revealed a positive association between gratitude and life satisfaction, and social support was a partial mediator in this relationship. Although enjoyment of life was not a direct mediator in the relationship between gratitude and life satisfaction, the final model indicated a significant serial pathway from gratitude to social support and then through enjoyment of life to life satisfaction. In conclusion, enhancing gratitude, promoting the enjoyment of life, and social support to older individuals might increase their life satisfaction, which in turn might contribute to their successful aging.

## 1. Introduction

The world is aging very rapidly due to increased life expectancy and a decline in rates of fertility. According to a recent United Nations report, it is expected that in the next three decades, the number of people aged 65 and over will double globally, shifting from 9.3% of the population in 2020 to 16% in 2050 [1]. In Saudi Arabia, people aged 65 and over will represent 18.4% of the entire population [2]. The aging process is usually associated with various physical and psychosocial concerns [3]. It is therefore crucial to investigate factors associated with life satisfaction among older adults. Being satisfied with life for an older adult implies adapting to these age-related changes, thereby maintaining a good quality of life. Given that Saudi Arabia’s population is aging rapidly, there is a growing demand for psychological research focusing on older adults in this population [4]. Specifically, it would be important to identify factors that enhance the aging experience in Saudi Arabia.

The socioemotional selectivity theory postulates that the aging process provides older people with a greater awareness that one’s lifetime is limited, which motivates them to pursue goals that benefit them in the present [5]; thus, for example, older adults would invest much of their time and energy in making and maintaining healthy social relationships with people who are meaningful to them. With such social bonds comes greater social support, which has been found to yield positive effects on subjective well-being and life satisfaction among older adults [6]. Other prior research has also examined the benefits of healthy social relationships for well-being in old age, as well as the relationship of gratitude with well-being. For example, Killen and Macaskill reported that expressing gratitude contributed to increases in subjective well-being in older people [7]. Similarly, people who are grateful also perceive greater social support [8] and enjoyment of the present [9]. These findings may be sensitive to cultural differences, indicating the need for research from other cultures.

Most studies have been conducted in individualistic cultures and few have been conducted in collectivistic cultures [10,11,12]. People from these cultures think differently about themselves. For example, someone from an individualistic culture derives happiness from the achievement of personal goals while someone from a collectivistic culture derives happiness from social harmony [13,14,15,16]. Saudi Arabia belongs also to this collectivistic culture, where people are not concerned with individual standpoints but rather group cohesion [17]. Another difference is the expression of emotions. People from individualistic cultures tend to experience either positive or negative emotions while people from collectivistic cultures tend to experience positive and negative emotions simultaneously [18,19], especially when it comes to receiving a favor. For example, a study conducted in the Japanese culture, a collectivistic culture, reported that gratitude was expressed with positive (warmth) and negative feelings (regret, indebtedness, and remorse) [20]. This difference in the expression of emotions can have an impact on the relationship between gratitude and other outcomes.

There is limited evidence of the association between gratitude and life satisfaction in older people in Saudi Arabia. One study investigated the relationship of gratitude with life satisfaction and the mediation role of perceived stress [21]. It was found that gratitude was positively related to life satisfaction and that this relationship was fully mediated by perceived stress; however, this research studied undergraduate students. To the best of our knowledge, no study has investigated the relationship between gratitude and life satisfaction and the mediation role of social support and enjoyment of life among older adults in Saudi Arabia. The purpose of this current study was, therefore, to contribute to the literature by examining the associations between these psychological variables and to disentangle the hypothesized mediating role of social support and enjoyment of life in older individuals in Saudi Arabia.

### 1.1. Gratitude and Life Satisfaction

Gratitude has been found to be associated with multiple positive life outcomes. In a comparative mixed-sample study involving middle-aged and older adults in the United States and Japan, positive associations between gratitude, relationship satisfaction, and overall life satisfaction were found [12]. Others have argued for a reciprocal relationship. A longitudinal design study with a sample of Latin Americans by Unanue and colleagues reported the existence of a positive reciprocal relationship between gratitude and life satisfaction [22], indicating that gratitude increases life satisfaction and that, in turn, life satisfaction enhances gratitude over time.

Intervention studies have also supported this relationship. Killen and Macaskill conducted a study involving a two-week intervention with the purpose of increasing gratitude in older adults [7]. The results of their study showed that the gratitude-enhancing intervention resulted in an increased sense of well-being and happiness and a decrease in perceived stress. Similarly, it has been established that gratitude training was better at enhancing older people’s well-being than optimism training [23]. Their findings indicated that the gratitude training intervention increased not only life satisfaction but also happiness and resilience. These effects were also found following an intervention consisting of writing gratitude letters, resulting in positive affect increases over the course of three weeks [24]. Gratitude journaling, another type of gratitude practice, was also found to be effective in enhancing positive affect in comparison to a control group [25]. In another randomized controlled trial of 16 weeks, it was found that an Islamic secular approach to expressing gratitude enhanced happiness [26]. A study by Rash and colleagues reported that a gratitude intervention benefited respondents with low trait gratitude in increasing life satisfaction [27]. These studies indicate positive benefits of gratitude for individuals’ subjective well-being; however, the findings are mostly from studies conducted in the West, and further research is therefore needed from other parts of the world.

Gratitude interventions were also found to enhance other psychological variables. In a sample of undergraduate students using a quasi-experimental design, Flinchbaugh and colleagues found that gratitude journaling training coupled with stress management training increased participants’ sense of meaning in their lives [28]. Another randomized controlled trial claimed that gratitude journaling training resulted in an increase in optimism [29]. Similarly, it was found in a two-week randomized controlled trial that a gratitude intervention increased hedonic well-being, optimism, and sleep quality compared to a control group [30]. The fulfillment of psychological needs has been also shown to benefit from gratitude interventions. For example, in a prospective study, Lee and colleagues found that gratitude predicted participants’ relatedness and autonomy [31]. All these findings, despite the different methodologies used, highlight the positive contribution of gratitude in people’s lives; however, these studies were conducted with samples of young people, indicating the need for research in old age.

### 1.2. Social Support and Enjoyment of Life as Mediators

Social support has long been thought to be a protective factor for well-being. A study showed that various sources of social support were predictive of emotional well-being among older adults in China [32]. Social support was also found to be associated with life satisfaction in Chinese empty nesters but this relationship was mediated by loneliness [33].

Social support has also received attention as a mediating agent. For example, social support acts as a mediator between forgiveness and life satisfaction. Different studies supported social support as a mediator. Zhu investigated the mediation role of social support in the relationship between forgiveness and life satisfaction and found that forgiveness was related to life satisfaction through social support [34]. Another study reported that gratitude was linked to life satisfaction through the paths of social support and self-esteem among adolescent survivors of a disaster [35]. Social support also mediated the association between gratitude and life satisfaction and between gratitude and burnout among student-athletes in the United States [36]. Nevertheless, there is a lack of evidence of the mediation role of social support in the association between gratitude and life satisfaction in older adults, particularly in the aging population in Saudi Arabia.

It has been argued that gratitude maximizes the enjoyment of pleasurable experiences [37] and facilitates relaxation and enjoyment of life [9]. In turn, previous studies reported that enjoyment of life was associated with life satisfaction among older adults. A study from Britain engaging not less than 999 individuals, aged 65 and over, reported that among the activities that enhanced the quality of life of respondents, participating in hobbies and leisure activities and having enough money to enjoy life emerged as important factors [38]. Smith and colleagues reported that, in a sample of older individuals, sexual activity was linked to enjoyment of life, which in turn increased life satisfaction [39]. The study reported that the well-being of older adults increased when they were sexually active. This relationship was also reported in a longitudinal study involving individuals aged 50 and over in England. They reported that reduced life enjoyment was related to higher mortality rates [40]. These results imply the converse, namely that enjoyment of life might reduce rates of mortality in old age. Using nationally representative data, Steptoe and Wardle found that there is an association between enjoyment of life and survival in old age [41]. They concluded that enjoyment of life can enhance subjective well-being in old age. In addition, it was found in a sample of individuals aged 60 and over that low levels of enjoyment of life in old age may be associated with future disability [42].

**Figure 1:**
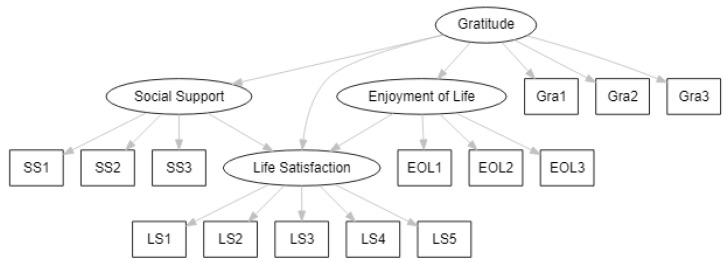
Conceptual model.

With increased socio-economic growth in recent years in Saudi Arabia, there have been changes in the lifestyle of Saudi Arabians. Research is therefore needed to address how the manner in which elderly Saudi Arabians try to enjoy life contributes to their sense of life satisfaction. The aim of this study was, therefore, to investigate the relationship between gratitude and life satisfaction, and the mediating roles of social support and enjoyment of life in a sample of older adults in Saudi Arabia. The conceptual framework is plotted in Figure 1. Accordingly, we formulate these hypotheses:

**Hypothesis** **1.** *Gratitude will have a positive association with life satisfaction among older adults*.

**Hypothesis** **2.** *Social support will mediate the relationship between gratitude and life satisfaction*.

**Hypothesis** **3.** *Enjoyment of life will mediate the relationship between gratitude and life satisfaction*.

## 2. Materials and Methods

### 2.1. Participants and Procedure

This cross-sectional study used a sample of older adults residing with their families and aged 60 years and over, according to the definition by the World Health Organization of older adults. The sample was determined using convenience sampling methods. After excluding all individuals less than 60 years of age, cases with missing data, and those residing in nursing homes, a sample of 260 older individuals aged 60 to 80 was yielded. The data were collected through self-reported questionnaires. A link to the Google Forms questionnaire was sent to participants by email or on social media, including Facebook and Twitter. Among the 260 respondents, 54.6% were females and 45.5% were males, and the mean age was 65.3. The respondents were informed about the study and they provided consent to participate.

### 2.2. Measures

The questionnaire consisted of several scales that have demonstrated good validity and reliability in the literature.

The Gratitude Resentment and Appreciation Test (GRAT) [43] was used to measure gratitude. The GRAT consists of forty-four items, with responses on a nine-point Likert scale from one (I strongly disagree with the statement) to nine (I strongly agree with the statement). The scale has three subscales: sense of abundance (abbreviated Gra1 in this study); appreciation for simple pleasures (Gra2); and social appreciation (Gra3). This scale was found to have good factorial validity and internal consistency reliability [43]. The scale has been validated in Saudi Arabia [44]. The scale demonstrated good internal consistency validity in this study (Cronbach’s alpha = 0.89).

The Enjoyment of Life Scale [45] was used to measure enjoyment of life. This scale contains sixty items recorded using a three-point Likert scale: one (rarely), two (sometimes), and three (always). This scale has cognitive (EOL1), affective (EOL2), and socio-behavioral subscales (EOL3). Total scores ranging between 60 and 180 were used in the analysis. This scale demonstrated internal consistency reliability ranging between 0.82 and 0.87 (Abdel-Aal and Mazloum, 2013). In this study, the scale has an excellent Cronbach’s alpha value of 0.96.

The Multidimensional Scale of Perceived Social Support Scale (MSPSS) [46] was used to measure perceived social support. This scale has twelve items scored on a seven-point Likert scale ranging from one (very strongly disagree) to seven (very strongly agree) with three subscales: significant other subscale (SS1); family subscale (SS2); and friends subscale (SS3). The scale demonstrated good construct validity and internal reliability and test-retest reliability [46]. The scale had been adapted in Saudi Arabia [47]. The scale had a sufficient Cronbach’s alpha value of 0.85.

The Satisfaction with Life Scale [48] was used to measure life satisfaction. This scale consists of five items (LS2, LS2, LS3, LS4, LS5) scored on a seven-point Likert scale ranging from one (strongly disagree) to seven (strongly agree). This scale also exhibited high internal consistency and temporal reliability [48]. The scale had been adapted to the Arabic language [49]. The scale exhibited sufficient internal consistency reliability (Cronbach’s alpha = 0.80).

A set of sample characteristic variables was also collected in the questionnaire, including gender, age, marital status, employment status, living status, income, health status, and the number of children.

### 2.3. Statistical Analysis

The statistical analyses were conducted in three phases. The first phase consisted of descriptive statistics (Table 1 and Table 2). The second phase consisted of mediation analysis with structural and equation models (SEM) using the ‘lavaan’ package [50]. The third phase consisted of bootstrapping methods to test the mediation effect (Table 3). The data were managed and analyzed using the R statistical software package [51].

### 2.4. Ethical Consideration

The permissions to conduct this study were provided by the Deanship of Scientific Research (DSR) at King Abdulaziz University, Jeddah.

## 3. Results

### 3.1. Descriptive Statistics

The descriptive statistics for the variables are summarized in Table 1 and Table 2. Table 1 summarizes the categorical variables and the prevalence of life satisfaction per category while Table 2 summarizes the continuous variables and the bivariate Pearson correlation between the main variables.

### 3.2. Hypotheses Testing

To test the hypotheses, structural equation models were employed. Firstly, two models were analyzed: a fully mediated model (Model 1) in which gratitude and life satisfaction were only associated indirectly through social support and enjoyment of life; a partially mediated model (Model 2) with indirect paths and a direct path from gratitude to life satisfaction. An ANOVA test indicated a significant chi-square difference between the two models (∆ꭓ^2^ = 79.98, *p* < 0.001). This shows that Model 2 (ꭓ^2^(60) = 210.04, *p* < 0.001; RMSEA = 0.09; SRMR = 0.11; CFI = 0.90; TLI = 0.87) better fit the data than Model 1 (ꭓ^2^(62) = 289.82, *p* < 0.001; RMSEA = 0.11; SRMR = 0.18; CFI = 0.85; TLI = 0.81).

**Figure 2:**
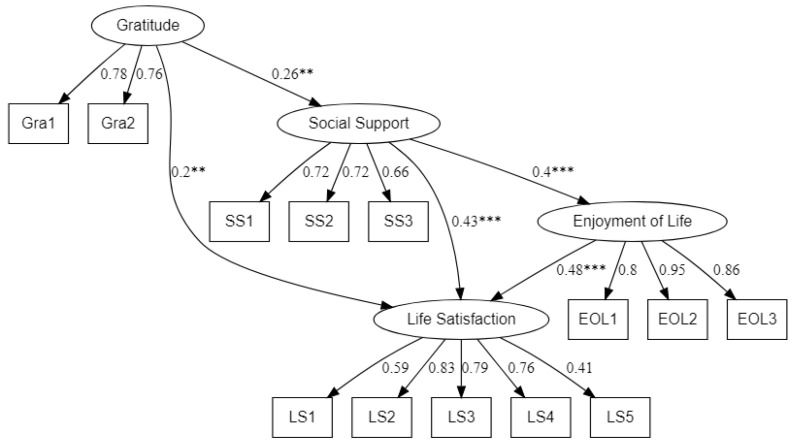
The final mediation model. Note: ** *p* < 0.01; *** *p* < 0.001.

However, the social appreciation subscale poorly loaded to the gratitude scale, so it was deleted in Model 3. In addition, the path from gratitude to enjoyment of life was not significant; therefore, in order to improve the model, this path was deleted and another path from social support to enjoyment of life was added. The results indicated that Model 3 better fit the data (ꭓ^2^(60) = 179.13, *p* < 0.001; RMSEA = 0.08; SRMR = 0.06; CFI = 0.92; TLI = 0.90). Thus, Model 3 was taken as the final structural model and the paths are graphed using the ‘lavaanPlot’ package [52] see Figure 2.

The path from gratitude to life satisfaction was significant (β = 0.20, *p* < 0.01) and Hypothesis 1 was supported. Finally, as shown in Table 3 and Figure 2, social support and enjoyment of life mediated the relationship between gratitude and life satisfaction. Although enjoyment of life mediated the relationship between gratitude and life satisfaction only via social support, Hypotheses 2 and 3 were supported.

## 4. Discussion

The purpose of this present study was to examine the association between gratitude and life satisfaction and to test the hypothesized mediation role of social support and enjoyment of life in the said relationship in a sample of older adults in Saudi Arabia. The results showed that gratitude was related to the life satisfaction of older individuals. This means that those who expressed greater gratitude reported increased satisfaction with life beyond the contributions of confounding variables. This corroborates prior studies that have found similar trends in older individuals [7,38].

Gratitude benefits life satisfaction through several mechanisms. Wood and colleagues postulated that individuals with increased levels of gratitude perceive help as costly, valuable, and altruistic [53]. They concluded that, unlike ungrateful people, grateful people view help as beneficial for them and consequently appreciate the supportiveness of their social connections. A second mechanism may be the positive coping strategies associated with expressing gratitude. A study investigated the links between gratitude and coping strategies [54]. They claimed that people with high gratitude also avail themselves of instrumental and emotional social support; moreover, their study concluded that grateful people were more likely to engage in active coping, planning, and positive reinterpreting of situations and were less likely to disengage and deny the existence of a problem or escape through substance use. Another explanation is that gratitude provides an antidote to stressful situations by helping people to develop resilience [55]. Expressing gratitude has also been linked to the ability to savor positive situations, which increases life satisfaction [56]. Finally, in times of high gratitude, negative emotions are inhibited [57]. The results of this study join those of previous research in encouraging gratitude as a low-cost treatment [58,59,60] and a non-pharmacological treatment [61] that can improve health in the population with little expenditure.

The positive associations found between enjoyment of life, social support, and life satisfaction are easily understandable. Enjoyment of life implies the experience of positive emotions. This is particularly important in later life in order to buffer the negative effects of age-related factors. It was found in the elderly that the more they engaged in leisure activities, the more likely they were to report high levels of subjective well-being [62]. Similarly, in a sample of retirees, it was reported that having fun in leisure activities was associated with increased levels of life satisfaction, and consequently with successful adaptation to retirement [63]. In another study, leisure activities were found to yield health benefits, thereby moderating the effects of stress [64]. Enjoyment of life was also associated with mortality rates: those who enjoyed life were more likely to live longer [41]. Social support also yields health benefits, especially in old age. A study by Penninx and colleagues found that high levels of social support were associated with a reduced risk of mortality [65]. In another sample of individuals aged 65 and over, it was found that social support mitigated depressive symptoms [66,67], thereby enhancing subjective well-being. All these findings explain the positive relationships found between enjoyment of life, social support, and life satisfaction in old age.

This study found that social support mediated the relationship between gratitude and life satisfaction among older adults. These results corroborate those of prior studies that found similar patterns in young individuals [34,35,36]. This study provides external validity for prior studies by revealing this association in a sample of older adults from another culture. Enjoyment of life failed to directly mediate the relationship between gratitude and life satisfaction as the path from gratitude to enjoyment of life was not significant. The mechanisms underlying this finding are to be found in the cultural patterns of Saudi Arabia; with Saudi Arabia being a collectivistic culture, it is possible that expressing gratitude may be accompanied by both positive and negative emotions—such as regret, indebtedness, and remorse—as it was found in collectivistic cultures [68,69]. This may explain why expressing gratitude may not directly lead to feelings of enjoyment but it seems that it does so through social support. To the best of our knowledge, this study is the first that examined the sequential mediation pathway from gratitude to social support, to enjoyment of life, and then to life satisfaction among older adults.

## 5. Conclusions

This study presents a picture of the relationships between gratitude and life satisfaction and the mediation role of social support and enjoyment of life in older individuals in Saudi Arabia. The main findings revealed a positive association between gratitude and life satisfaction. Moreover, a mediation role of social support and enjoyment of life was revealed in a serial pathway model. These findings are relevant to elderly individuals, to health care systems, and to the field of geriatrics in general. Efforts to enhance gratitude, social support, and enjoyment in old age can increase life satisfaction, which might contribute to successful aging. In fact, interventions to enhance gratitude have been found to be successful and programs to engage older adults in enjoyable activities might bring similar benefits. For practitioners, these findings can inform which interventions should be used to promote the life satisfaction of older adults in a country that is expected to have an increased number of older adults. For future research, it is important to address these associations using longitudinal designs in order to obtain additional insights; moreover, randomized controlled trials among older adults in Saudi Arabia might yield better insights than observational studies.

Despite the implications of this study, there are some limitations that must be acknowledged. First, the design of the study was cross-sectional. Therefore, no causal effects can be assumed. Future research should use longitudinal designs in order to determine causality. Second, the data were collected via self-reported questionnaires. Future research should use other methods of data collection, including interviews. Third, the study used convenience sampling methods, which are not ideal for the generalizability of the findings. Future research should use random sampling.

## Figures and Tables

**Table 1:** Descriptive statistics of categorical variables and the prevalence of LS per category.

Variable		n	%	Mean of LS	SD of LS
Gender		260			
	Female	142	54.6	22.7	6.40
	Male	118	45.4	23.5	4.70
Marital Status		260			
	Married	212	81.5	23.9	4.97
	Unmarried	48	18.5	19.6	7.26
Living Status		260			
	Alone	12	4.6	15.5	5.90
	With a son	12	4.6	24.1	7.44
	With family	236	90.8	23.4	5.33
Income		260			
	SAR <5000	18	6.9	20.33	5.15
	SAR 5000–10000	220	84.6	23.01	5.80
	SAR >10000	22	8.5	26.54	3.09
Health Status		260			
	Poor health	112	43	22.42	6.40
	Good health	148	57	23.66	5.06
Employment status		260			
	Employed	86	33.08	22.34	5.33
	Retired	108	40.77	22.92	5.67
	Unemployed	68	26.15	24.44	6.04
Number of children					
	Less than 5	94	36.15	22.93	5.00
	5 or more	166	63.85	23.24	6.07

Note: SD = Standard Deviation; LS = Life Satisfaction (range = 5–35).

**Table 2:** Descriptive statistics of continuous variables and the bivariate correlations.

Variable	n	Mean	SD	1	2	3	4
Gratitude	260	89.38	10.42	1			
Social Support	260	61.24	11.22	0.06	1		
Life Satisfaction	260	23.13	5.70	0.16 **	0.44 ***	1	
Enjoyment of Life	260	150.6	19.62	−0.12 *	0.29 ***	0.59 ***	1

Note: * *p* < 0.05; ** *p* < 0.01; *** *p* < 0.001.

**Table 3:** Indirect effects in Model 3 and 95% confidence intervals.

Model Pathways	Estimate Effect	95% CI	
		Lower	Upper
Gratitude→Social Support→Life Satisfaction	0.020 **	0.006	0.035
Gratitude→Social Support→Enjoyment of Life→Life Satisfaction	0.010 *	0.002	0.016

Note: * *p* < 0.05; ** *p* < 0.01.

## Data Availability

The data that support the findings of this study are available from the corresponding author upon reasonable request.
